# Aortic pulse wave analysis and functional capacity of heart transplantation candidates: a pilot study

**DOI:** 10.1038/s41598-024-61152-w

**Published:** 2024-05-07

**Authors:** Adriana Marques Alcici-Moreira, Marcela Oliveira Vitarelli, Tiago Abreu Velloso, Igor Antônio Carvalho-Ribeiro, Daniella Moura Dario, Janaine Cunha Polese, Hélio Penna Guimarães, José Luiz Barros Pena, Marcelo Tuesta, Bruno Almeida de Rezende, Maria da Glória Rodrigues-Machado

**Affiliations:** 1 Post-Graduate Program in Health Sciences of Medical Sciences Faculty of Minas Gerais (FCM-MG), Alameda Ezequiel Dias, 275, Belo Horizonte, MG 30130-110 Brazil; 2 Federal University of Minas Gerais, Belo Horizonte, MG Brazil; 3 Hospital Israelita Albert Einstein, São Paulo, SP Brazil; 4 Exercise and Rehabilitation Sciences Laboratory, School of Physical Therapy, Faculty of Rehabilitation Sciences, Universidad Andres Bello, Santiago, Chile

**Keywords:** Heart transplantation, Heart failure, Vascular stiffness, Pulse wave analysis, Metabolic equivalent, Cardiology, Interventional cardiology

## Abstract

We compared cardiovascular parameters obtained with the Mobil-O-Graph and functional capacity assessed by the Duke Activity Status Index (DASI) before and after Heart Transplantation (HT) and also compared the cardiovascular parameters and the functional capacity of candidates for HT with a control group. Peripheral and central vascular pressures increased after surgery. Similar results were observed in cardiac output and pulse wave velocity. The significant increase in left ventricular ejection fraction (LVEF) postoperatively was not followed by an increase in the functional capacity. 24 candidates for HT and 24 controls were also compared. Functional capacity was significantly lower in the HT candidates compared to controls. Stroke volume, systolic, diastolic, and pulse pressure measured peripherally and centrally were lower in the HT candidates when compared to controls. Despite the significant increase in peripheral and central blood pressures after surgery, the patients were normotensive. The 143.85% increase in LVEF in the postoperative period was not able to positively affect functional capacity. Furthermore, the lower values of LVEF, systolic volume, central and peripheral arterial pressures in the candidates for HT are consistent with the characteristics signs of advanced heart failure, negatively impacting functional capacity, as observed by the lower DASI score.

## Introduction

Heart failure is a complex multifactorial syndrome, whose main risk factors are chronic ischemic heart disease, systemic arterial hypertension, valvular and rheumatic disease, idiopathic dilated cardiomyopathy, chagasic cardiomyopathy, cardiomyopathy induced by chemotherapy and radiotherapy, also congenital heart disease^1^. Heart failure affects more than 64 million people worldwide^1^. In Brazil, the prevalence is approximately two million patients, and its incidence is 240,000 new cases per year^2^.

Several classification systems have been developed to characterize patients with heart failure and define those with advanced disease. The classification of New York Heart Association categorizes the relationship between symptoms of dyspnea and physical activity into 4 classes, ranging from no symptoms and normal physical activity (class I) to severe dyspnea at rest (class IV)^3^. Advanced heart failure is a clinically important designation as it can direct advanced therapies including heart transplantation and mechanical ventricular assist. According to the European Society of Cardiology, this classification includes patients from classes III or IV, ejection fraction ≤ 30%, right ventricular dysfunction, inoperable congenital heart or valve abnormalities, pulmonary congestion or low cardiac output requiring intravenous diuretics or inotropes, and severe reduction in exercise capacity of cardiac origin^4^.

Heart transplantation is a therapeutic option that prolongs the lives of patients with advanced heart failure, also improves functional capacity and quality of life^5^. Cardiopulmonary exercise testing is considered the gold standard for assessing functional capacity. Although, its high financial cost makes it inaccessible to most patients^6^. Another frequently used metric is the 6-min walk test, which is a measure of functional capacity that reflects exercise performance and the patient's ability to perform activities of daily living. The distance covered in 6 min is strongly related to the maximum oxygen consumption (VO_2_ max)^4^. Given the impossibility of using tests, scales, and questionnaires that assess functional capacity, are being increasingly used in clinical practice. Among the available options, the self-administered questionnaire stands out Duke Activity Status Index (DASI), designed to measure the functional capacity and also assess aspects of the quality of life of patients with cardiovascular diseases, based on the patient's ability to perform a set of common activities of daily living^7,8^.

Study shows that DASI score can be used to get a rough estimate of patients’ peak oxygen uptake (peakVO_2_) in patients with cardiovascular disease^9^; provides information on disease severity, rehabilitation effects^10^, and prognosis in patients with cardiovascular diseases. The score of DASI ranges from 0 to 58.2 ml kg^−1^ min^−1^, and higher scores indicate better functional capacity. According to Grodin et al.^11^, the score of 26.2 ml kg^−1^ min^−1^ was able to predict the 5-year mortality of patients with heart failure.

Increased arterial stiffness is commonly used as an indicator of cardiovascular risk and has been directly associated with a worse cardiovascular prognosis in various populations and ages, regardless of other risk factors^12,13^. Souza-Neto et al. demonstrated that arterial hypertension was independently associated with the estimation of arterial stiffness in a cohort of adult patients who received heart transplantation. Renal dysfunction, diabetes, and lack of nocturnal diastolic descent have been identified as determinants of arterial stiffness^14^.

Until now, to our knowledge, no study has simultaneously evaluated vascular pressures, hemodynamic parameters, and arterial stiffness indices in patients undergoing heart transplantation. Therefore, this study aims to verify the impact of heart transplantation on cardiovascular parameters and functional capacity, assessed by DASI, and to compare cardiovascular parameters and functional capacity of patients who are candidates for heart transplantation with the control group, matched by sex and age.

## Methods

This is a pilot study with a mixed design, which involved data collection for cross-sectional and longitudinal studies. The longitudinal study compared cardiovascular parameters and functional capacity, through the questionnaire DASI, before and after heart transplantation in patients treated at a philanthropic hospital, from April 2022 to May 2023. The cross-sectional study compared cardiovascular parameters and functional capacity of candidates for heart transplantation, in baseline conditions, with a control group composed of individuals considered healthy, matched by sex and age. This study was approved by the Research Ethics Committee at *Faculdade Ciências Médicas de Minas Gerais* and by the Research Ethics Committee represented by Dr. Francisco das Chagas Lima e Silva—*Santa Casa de Misericórdia de Belo Horizonte* (number: 5.111.195). All participants signed an informed consent form.

### Study population

Candidates for heart transplantation of both sexes, aged over 18 years, who had clinical stability without the use of vasoactive drugs at the time of evaluation, were included. Patients diagnosed with chronic kidney disease and/or candidates for double transplantation (heart-kidney) and those receiving intravenous vasodilators at the time of evaluation were excluded. The control group consisted of volunteers considered healthy, recruited through an active search in the general community, and matched by sex and age.

### Clinical and anthropometric evaluation

Clinical and laboratory data were extracted from the patient’s charts. The collection of laboratory data coincided with the assessment of cardiovascular parameters. The evaluated comorbidities were diabetes mellitus, systemic arterial hypertension, and dyslipidemia, in addition to the etiology of heart failure. Diabetes mellitus was defined by fasting blood glucose ≥ 126 mg/dL or the use of insulin or oral hypoglycemic agents. Hypertension was explained as systolic blood pressure ≥ 140 mmHg or diastolic ≥ 90 mmHg. Dyslipidemia was set out as total serum cholesterol above 200 mg/dL or use of statins^15^.

Past history of coronary interventions or acute myocardial infarction and of stroke or transient ischemic attack were considered for the analysis. The registration of continuous-use medications was carried out.

The body mass index (BMI) was calculated from the ratio between the individual's weight (kg) and height (m^2^). Body surface area was estimated by electronic calculation based on the formula of Du Bois and Du Bois^16^.

### Echocardiogram

Transthoracic echocardiography was performed in accordance with the joint recommendations of the American Society of Echocardiography and the European Association for Cardiovascular Imaging^17^. The equipments used were GE Vivid T8 Ultrasound System (General Eletric Company Inc. GE Medical Systems—China) or Ultrasound Affiniti 50 (Philips Ultrasound, Inc.—EUA). Echocardiographic studies were performed using M-mode, two-dimensional, pulsed and color Doppler, by parasternal, subcostal, apical and suprasternal acoustic windows.

### Functional class of heart failure

The scale of the *New York Heart Association* (NYHA) was used to determine the functional class of patients with heart failure. The class NYHA consists of a stratum of 4 levels, ranging from class I to class III is characterized by significant limitations in physical activity, with comfort at rest; however, activities that are less intense than usual cause symptoms of heart failure. Class IV denotes worse functional status, that is, the presence of symptoms of heart failure even at rest^18^.

### Assessment of cardiovascular indices

Cardiovascular indices were obtained by the equipment Mobil-O-Graph (IEM, Stolberg, Germany), validated in comparative studies with invasive methods^19^ and not invasive^20^. Central arterial pressures were estimated non-invasively from brachial pulse oscillometry. For this, we used the algorithm ARCSolver (Austrian Institute of Technology, Vienna) and a high-fidelity sensor (MPX5050; Freescale, Tempe, AZ, EUA) incorporated into the cuff of the device^21^. In order to choose the appropriate cuff, the arm circumference was measured and the cuff was positioned two centimeters above the cubital fossa.

The arterial stiffness indices evaluated were the carotid-femoral pulse wave velocity (PWV), considered the gold standard to determine this alteration, the augmentation index (AIx), and augmentation pressure (PAo). The high-fidelity cuff measures peripheral blood pressure, then re-inflates at the diastolic blood pressure level for ten seconds to capture pulse wave information generated in the brachial artery. Using a mathematical model, the equipment program generates the aortic pulse wave, estimating central pressures and PWV, and separates the waves from the decomposition of the aortic pulse wave into ejection wave and reflected wave^21,22^. The PWV expresses the ratio between distance and time in which the pulse is transmitted between the carotid and femoral arteries after cardiac ejection, being calculated by Δx/Δt^23,24^.

AIx@75 is calculated from the aortic pulse wave by means of the pressure difference between the peak of the reflection wave (P2) and the peak of the incident wave (P1), expressed as a percentage of the central pulse pressure (cPP). The difference between P1 and P2 corresponds to PAo, which measures the absolute elevation of central systolic blood pressure due to the early return of the reflected wave. In this way, AIx@75 is expressed by the ratio between the PAo/cPP × 100^23^.

In addition to arterial stiffness indices, peripheral arterial pressures were evaluated [systolic blood pressure (pSBP), diastolic blood pressure (pDBP), pulse pressure (pPP)] and central (cSBP, cDBP, cPP), and hemodynamic parameters: stroke volume (SV), cardiac output (CO), total vascular resistance (TVR), cardiac index (CI) and heart rate (HR). Measurements were performed in triplicate and the average was considered for the final analysis.

The software ARCSolver includes an algorithm that checks the signal quality on a graduated scale from 1 to 5. In this study, only signal quality in grades 1 and 2 was considered, which represent very good or good quality, respectively, producing reliable data. It means that during signal acquisition, more than 80% (quality: 1) and more than 50% (quality: 2) of cardiac cycles were recorded.

Cardiovascular parameters were collected in two moments: preoperatively (after hospitalization and surgical decision) and intermediate postoperative period (30 to 60 days after surgery in an outpatient unit), corresponding to 43.8 ± 14.05 days after transplant. Control group volunteers were evaluated at *Faculdade Ciências Médicas de Minas Gerais*.

### Assessment of functional capacity

The Duke Activity Status Index (DASI) assessed functional capacity. It is a self-administered questionnaire, consisting of 12 items that describe activities of daily living. The research subjects answer whether or not they are able to perform activities such as: personal care, ambulation, housework, sexual function, and recreational activities. Each item has a specific weight, according to the metabolic cost (MET) of each activity. The score ranges from 0 to 58.2 ml kg^−1^ min^−1^, and higher scores indicate better functional capacity. The instrument was translated and cross-culturally adapted for the assessment of Brazilian individuals with cardiovascular diseases^25^. The application of the questionnaire was performed concurrently with the evaluation of cardiovascular parameters, in the moments: preoperative and intermediate postoperative period. In the control group, the DASI was applied only once.

### Statistical analysis

Qualitative variables were presented as absolute frequencies and their respective percentages, and quantitative variables as mean ± standard deviation [median]. The normality of the data was verified by the test of Shapiro–Wilk*.* For the comparison between pre and postoperative results, the paired t-test was used for parametric data and the Wilcoxon test for non-parametric data. In the comparison between the study group and the control group, the unpaired t-test or the Mann–Whitney test, when applied, was used. Effect size was calculated with Cohen’s d. The interpretation guideline used was 0.1–0.3 (small effect), 0.3–0.5 (moderate effect), and > 0.5 (large effect).

To assess BMI controlled effect of vascular, hemodynamic and arterial stiffness parameters on pre-transplant and control groups, binary logistic models were built, and the results showed as odds ratio (OR) and 95% confidence interval (CI).

For correlation analysis, Pearson's correlation coefficient was used for parametric data or Spearman's for non-parametric data. In all tests, the significance level adopted was 5%. Data analysis was performed using the software Prism 8 (GraphPad Software, Inc.).

### Ethics approval

This study was performed in line with the principles of the Declaration of Helsinki. Approval was granted by the Research Ethics Committee of the Faculty of Medical Sciences of Minas Gerais (protocol n. 48127521.6.3001.5138, approval report n. 5.111.195).

### Consent to participate

All participants signed an informed consent form.

## Results

**Figure 1:**
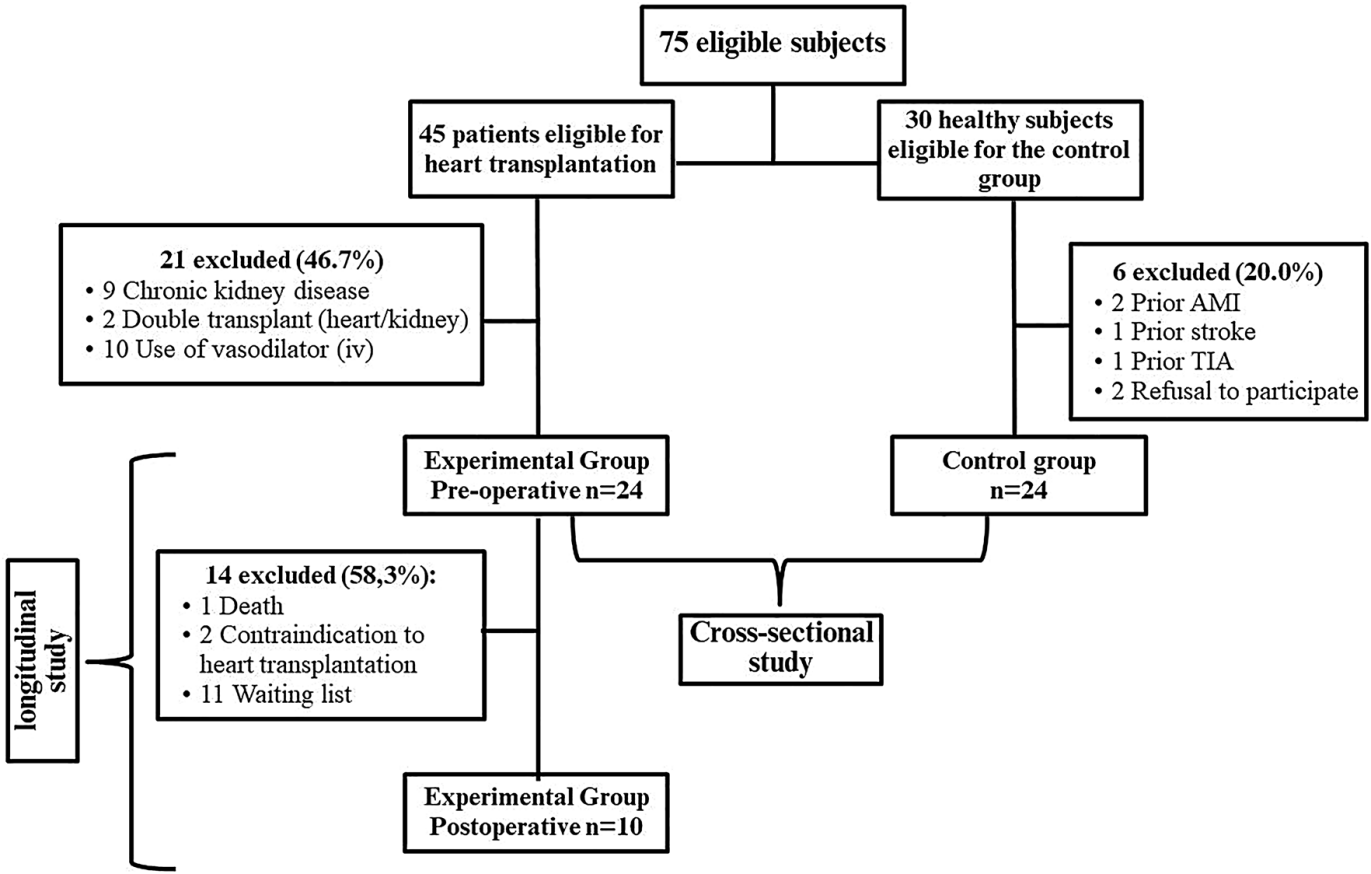
Selection of participants for the control group and heart transplantation groups. *AMI* Acute myocardial infarction. *TIA* Transient ischemic attack.

Of the 45 eligible patients, 21 were excluded because they had chronic kidney disease (n = 9), double heart-kidney transplantation (n = 2), and use of intravenous vasodilator (n = 10). Of the 24 patients who participated in the first collection, only 10 participated in the second collection. One patient died in the postoperative period, two patients did not undergo heart transplantation due to the severity of the clinical condition and eleven are still on the waiting list for the surgical procedure (Fig. 1).

The sample consisted of predominantly male individuals (70.8%) in both groups. The mean age was 50.17 ± 12.39 [53.00] years in the heart transplantation candidates and 51.04 ± 13.14 [53.50] years in the control group, without statistical significance.

Regarding the diagnosis, the most prevalent was Chagas cardiomyopathy (29.2%), followed by 25.0% of idiopathic dilated cardiomyopathy. The drugs most commonly used by participants were diuretics (95.8%), beta-blockers (91.7%), and angiotensin-converting enzyme inhibitors (75.0%). LVEF (%), assessed preoperatively, did not correlate with any cardiovascular parameters. The group of heart transplantation candidates had a significantly lower BMI than the control group (*p* = 0.001) (Table 1).

**Table 1:** Sample characterization.

Variables	Control group N = 24	Transplant candidates group N = 24	*p* value
	Mean ± DP [median]	Mean ± DP [median]	
Age (years)	51.04 ± 13.14 [53.50]	50.17 ± 12.39 [53.00]	0.868^ M^
Weight (kg)	75.51 ± 15.92 [72.00]	68.27 ± 14.12 [67.90]	0.102^ T^
Height (m)	1.61 ± 0.07 [1.66]	1.69 ± 0.12 [1.69]	0.327^ T^
BMI (kg/m^2^)	26.89 ± 4.28 [25.40]	23.67 ± 4.36 [23.35]	**0.001**^**M**^
body surface (m^2^)	1.86 ± 0.22 [1.81]	1.78 ± 0.22 [1.80]	0.226^ T^
Sex n (%)			–
Male	17 **(**70.8%)	17 **(**70.8%)	
Female	7 **(**29.2%)	7 **(**29.2%)	
Diagnosis of cardiomyopathy n (%)			
Chagasic cardiomyopathy	–	7 (29.2%)	–
Idiopathic dilated cardiomyopathy	–	6 (25.0%)	–
Peripartum cardiomyopathy	–	3 (12.5%)	–
Ischemic cardiomyopathy	–	2 (8.3%)	–
HFrEF of valve etiology	–	2 (8.3%)	–
Inflammatory cardiomyopathy	–	1 (4.2%)	–
Non-compacted myocardium	–	1 (4.2%)	–
HFrEF of ethanoic etiology	–	1 (4.2%)	–
Congenital cardiomyopathy	–	1 (4.2%)	–
Echocardiogram			
LVEF (%)	–	26.74 ± 6.11 [28.25]	–
SPAP (mmHg)	–	41.02 ± 12.28 [40.00]	–
Medicines			
Diuretics	–	23 (95.8%)	–
Beta-blockers	–	22 (91.7%)	–
Angiotensin-converting enzyme inhibitors	–	18 (75.0%)	–
Digitalis	–	9 (37.5%)	–
Antidiabetics	–	7 (29.2%)	–
Comorbidities			
Dyslipidemia	–	7 (29.2%)	–
Acute myocardial infarction	–	5 (20.8%)	–
Diabetes mellitus	–	3 (12.5%)	–
Stroke	–	1 (4.2%)	–
Functional class (NYHA)			
Class III	–	17 (70.8%)	–
Class IV	–	7 (29.2%)	–

^M^Mann-Whitney test; ^T^Unpaired t-test. *BMI* body mass index, *HFrEF* heart failure with reduced ejection fraction, *LVEF* left ventricular ejection fraction, *SPAP* systolic pulmonary artery pressure *NYHA* New York Heart Association.

Significant values are in bold.

### Comparison of cardiovascular parameters and functional capacity between the pre and post-transplantation periods of the heart transplant group

The length of stay of patients undergoing heart transplantation was 37.2 ± 11.17 days, and the main postoperative complication recorded in the medical records was infection (70.0%).

LVEF significantly increased in the post-transplantation period (63.40 ± 4.10 [63.52] %) from baseline (26.00 ± 6.19 [28.25]%) (*p* < 0.001).

Table 2 shows comparisons of vascular and hemodynamic parameters and arterial stiffness indices pre- and post-transplantation of 10 patients undergoing surgery. The pSBP, pDBP, and MAP increased 30.04; 30.43 and 30.48%, respectively, after heart transplantation, with effect sizes (ES) of 1.26, 1.43 and 1.36, respectively. Similar results were observed in cSBP (31.36% and ES 1.23) and cDBP (28.97% and ES 1.41). As for hemodynamic parameters, cardiac output (20.00% and ES 1.71) and cardiac index (25.71% and ES 0.97) also showed a significant post-transplant increase. Arterial stiffness indices were similar in both periods, with the exception of PWV, which showed a significant increase after surgery (11.56% and ES 0.59). Δ PWV strongly positively correlated with Δ pPAM (r = 0.969, *p* < 0.001), Δ cSBP (r = 0.980, *p* < 0.001) and Δ systolic volume (r = 0.772, *p* = 0.015). Δ LVEF or Δ heart rate were not associated with Δ PWV.

**Table 2:** Comparison of vascular and hemodynamic parameters, and arterial stiffness indices before and after heart transplantation (n = 10).

Variables	Pre-transplant Mean ± SD [median]	Post-transplant Mean ± SD [median]	*P* value	Cohen’s d
Peripheral pressures				
pSBP (mmHg)	106.50 ± 20.89 [99.83]	128.50 ± 13.34 [129.83]	**0.012**^**T**^	1.26
pDBP (mmHg)	72.68 ± 12.40 [69.00]	89.47 ± 11.05 [90.00]	**0.010**^**T**^	1.43
MAP (mmHg)	88.13 ± 15.66 [82.00]	106.80 ± 11.47 [107.33]	**0.010**^**T**^	1.36
pPP (mmHg)	33.80 ± 12.62 [31.00]	39.17 ± 8.77 [38.17]	0.152^**T**^	
Central pressures				
cSBP (mmHg)	97.23 ± 19.40 [90.33]	117.30 ± 12.61 [118.67]	**0.019**^**T**^	1.23
cDBP (mmHg)	74.00 ± 12.60 [70.17]	90.83 ± 11.13 [90.50]	**0.010**^**T**^	1.41
cPP (mmHg)	23.20 ± 9.15 [20.50]	26.52 ± 6.78 [26.17]	0.288^**T**^	
Hemodynamic parameters				
SV (ml)	51.00 ± 9.71 [53.55]	54.27 ± 7.41 [54.93]	0.506^**T**^	
CO (l/min)	4.37 ± 0.47 [4.50]	5.22 ± 0.52 [5.40]	**0.005**^W^	1.71
TVR (s*mmHg/ml)	1.22 ± 0.16 [1.20]	1.23 ± 0.06 [1.22]	0.645^W^	
CI (l/min*1/m^2^)	2.60 ± 0.57 [2.45]	3.11 ± 0.47 [3.08]	**0.008**^**T**^	0.97
HR (bpm)	88.53 ± 18.09 [85.67]	98.37 ± 9.24 [100.83]	0.118^**T**^	
Arterial stiffness				
PAo (mmHg)	4.86 ± 4.82 [2.83]	4.53 ± 3.11 [3.50]	1.000^W^	
RC (%)	55.60 ± 17.74 [58.50]	57.40 ± 10.25 [59.83]	0.759^**T**^	
AIx@75 (%)	23.80 ± 9.34 [21.50]	28.13 ± 10.78 [24.00]	0.202^W^	
PWV (m/s)	6.49 ± 1.17 [6.32]	7.24 ± 1.36 [7.37]	**0.013**^**T**^	0.59
PPA	1.49 ± 0.31 [1.36]	1.50 ± 0.20 [1.47]	0.980^**T**^	

^T^T test; ^W^Wilcoxon test. *pSBP* peripheral systolic blood pressure, *pDBP* peripheral diastolic blood pressure, *MAP* mean arterial pressure, *pPP* peripheral pulse pressure, *cSBP* central systolic blood pressure, *cDBP* central diastolic blood pressure, *cPP* central pulse pressure, *SV* systolic volume, *CO* cardiac output, *TVR* total vascular resistance, *CI* cardiac index, *HR* heart rate, *PAo* augmentation pressure, *RC* reflection coefficient, *AIx@75* augmentation index corrected for a heart rate of 75 bpm, *PWV* pulse wave velocity, *PPA* pulse pressure amplification. Numbers in square brackets represent the median. Effect size was calculated with Cohen’s d.

Significant values are in bold.

**Figure 2:**
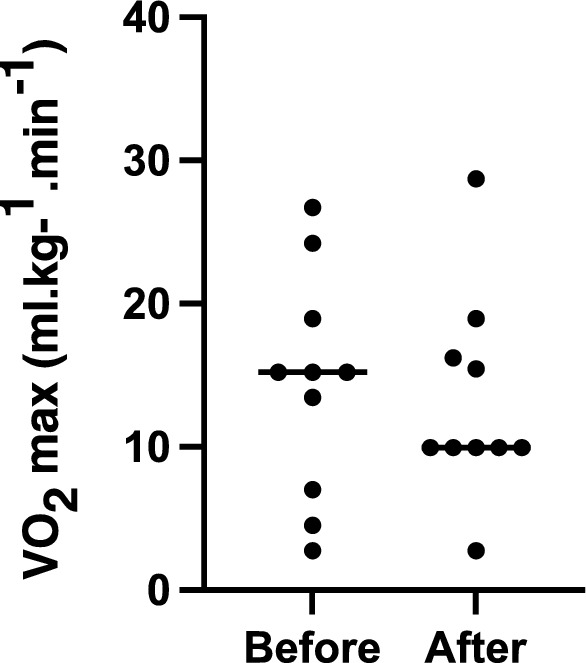
Comparison of the means of scoring Duke Activity Status Index (DASI) before and after heart transplantation. Paired t-test (*p* = 0.7675).

Figure 2 shows that the functional capacity assessed by the DASI was similar in the pre-(14.32 ± 7.88 [15.20] ml kg^−1^ min^−1^) and post-heart transplantation (13.18 ± 7.07 [9.95] ml kg^−1^ min^−1^).

### Comparison of cardiovascular parameters and functional capacity between the group of candidates for heart transplantation and the control group

The control group consisted of 24 volunteers considered healthy, paired by sex and age with the study group. Central and peripheral arterial pressures were significantly lower in the group of transplant candidates. Similar results were observed in hemodynamic parameters. Stroke volume was significantly lower and PPA was higher in the group of transplant candidates than the control group (Table 3). The effect controlled by BMI of vascular, hemodynamic and arterial stiffness parameters in the pre-transplant and control groups were carried out through the construction of binary logistic models and the results presented as odds ratio (OR) and confidence interval (CI) of 95% are shown in Table 3.

**Table 3:** Comparison of vascular, hemodynamic, and arterial stiffness parameters between pre-transplant and control groups.

Variables	Control group (n = 24)	Pre-transplant (n = 24)	OR (95%CI)	*P* value
Peripheral pressures				
pSBP (mmHg)	118.90 ± 8.65 [120.16]	103.80 ± 17.20 [100.83]	0.91 (0.85;0.96)	**0.002**
pDBP (mmHg)	78.72 ± 9.81 [78.83]	68.85 ± 11.99 [69.00]	0.92 (0.86;0.98)	**0.012**
MAP (mmHg)	94.84 ± 8.39 [96.50]	84.88 ± 13.89 [82.67]	0.92 (0.85;0.98)	**0.011**
pPP (mmHg)	40.18 ± 7.60 [40.67]	34.95 ± 9.32 [33.67]	0.92 (0.84;0.99)	**0.046**
Central pressures				
cSBP (mmHg)	111.00 ± 8.84 [111.00]	95.08 ± 16.08 [92.50]	0.90 (0.84;0.96)	**0.002**
cDBP (mmHg)	79.89 ± 9.66 [80.16]	69.92 ± 12.21 [70.17]	0.92 (0.85;0.98)	**0.011**
cPP (mmHg)	31.18 ± 6.34 [29.99]	25.19 ± 7.03 [25.67]	0.86 (0.76;0.95)	**0.010**
Hemodynamic parameters				
SV (ml)	68.64 ± 12.63 [69.26]	57.06 ± 14.94 [55.68]	0.95 (0.90;0.99)	**0.044**
CO (l/min)	4.88 ± 1.21 [4.97]	4.51 ± 0.80 [4.48]	0.54 (0.19;1.36)	0.218
TVR (s*mmHg/ml)	1.21 ± 0.14 [1.23]	1.16 ± 0.16 [1.16]	0.03 (0.00;1.61)	0.096
CI (l/min*1/m^2^)	2.63 ± 0.29 [2.60]	2.54 ± 0.51 [2.47]	0.22 (0.03;1.20)	0.104
HR (bpm)	72.39 ± 11.19 [71.50]	81.06 ± 17.01 [78.83]	1.04 (0.99;1.09)	0.140
Arterial stiffness				
PAo (mmHg)	6.22 ± 3.61 [5.50]	4.82 ± 3.47 [4.17]	0.85 (0.70;1.02)	0.093
RC (%)	64.44 ± 5.84 [65.33]	61.24 ± 13.12 [62.83]	0.99 (0.92;1.05)	0.730
AIx@75 (%)	18.78 ± 8.71 [19.67]	20.61 ± 10.02 [20.75]	0.98 (0.92;1.05)	0.621
PWV (m/s)	7.36 ± 1.38 [7.43]	6.69 ± 1.13 [6.47]	0.74 (0.43;1.22)	0.248
PPA	1.29 ± 0.10 [1.30]	1.40 ± 0.21 [1.41]	2.07 (4.24;1.06e+7)	**0.041**

Data presented as mean ± standard deviation [median]. *OR* odds ratio, *CI* confidence interval, *P* values and OR refers to binary logistic regression controlled by body mass index, *pSBP* peripheral systolic blood pressure, *pDBP* peripheral diastolic pressure, *MAP* mean arterial blood pressure, *pPP* peripheral pulse pressure, *cSBP* central systolic blood pressure, *cDBP* central diastolic blood pressure, *cPP* central pulse pressure, *SV* systolic volume, *CO* cardiac output, *TVR* total vascular resistance, *CI* cardiac index, *HR* heart rate, *PAo* augmentation pressure, *RC* reflection coefficient, *AIx@75* augmentation index corrected for a heart rate of 75 bpm, *PWV* pulse wave velocity, *PPA* pulse pressure amplification.

Significant values are in bold.

For pSBP (OR 0.91, *p* = 0.002), pDBP (OR 0.92, *p* = 0.012), MAP (OR 0.92, *p* = 0.011) and pPP (OR 0.92, *p* = 0.046) the increase of values reduces the chance to be from the pre-transplant group. Similar results were observed in cSBP (OR 0.90, *p* = 0.002), cDBP (OR 0.92, *p* = 0.011) and cPP (OR 0.86, *p* = 0.010), as well as for stroke volume (OR 0 0.95, *p* = 0.044). For PPA, the increase of values increases the chance to be from the pre-transplant group (OR 2.07, *p* = 0.041) (Table 3).

**Figure 3:**
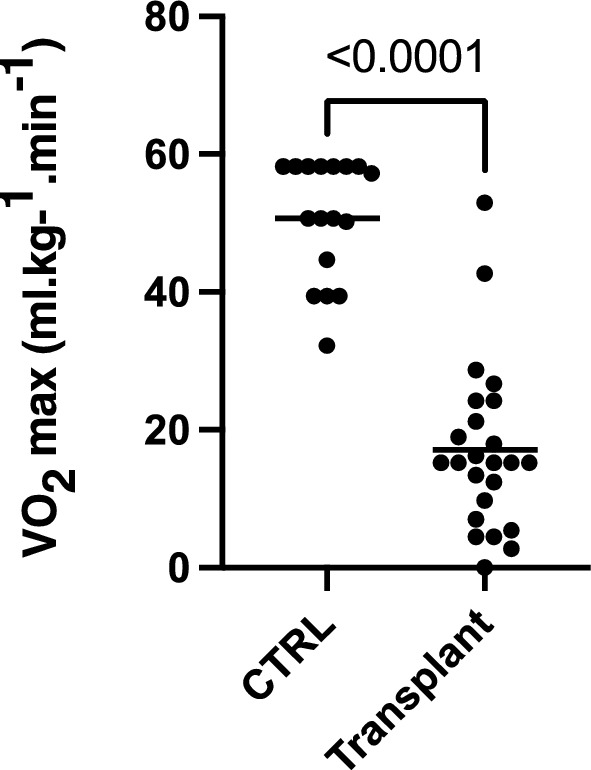
Comparison of score means of Duke Activity Status Index (DASI) between the control and heart transplant groups. Test of Mann–Whitney.

The DASI score was significantly lower in the transplant candidate group (17.07 ± 12.25 [15.20] ml kg^−1^ min^−1^) when compared to the control group (50.71 ± 8.64 [50.70] ml kg^−1^ min^−1^) (Fig. 3).

## Discussion

The present study showed that patients submitted to heart transplantation presented a significant increase in peripheral and central vascular pressures in the postoperative period; however, pressure levels were still within normal limits. The increase in PWV, also observed after transplantation, may have been modulated by an increase in mean arterial pressure, LVEF, or heart rate. The significant increase in LVEF in the post-transplant period was not accompanied by an increase in functional capacity assessed by the DASI. A comparison of cardiovascular variables assessed preoperatively and the control group showed that central and peripheral vascular pressures, systolic volume, and functional capacity were significantly lower in the group of transplant candidates.

### Comparison of vascular, hemodynamic, and arterial stiffness parameters before and after heart transplantation

In the present study, we observed that pSBP, pDBP, and MAP increased significantly in the postoperative period of heart transplantation. As this increase did not exceed the recommended limits for clinically characterizing arterial hypertension, these results suggest that heart transplantation promoted homeostasis in the cardiovascular system. Our results differ from some studies that show that arterial hypertension is one of the most common complications after heart transplantation, affecting up to 75% of patients at 1 year and 90% at 5 years after surgery^14,26,27^. This difference in results may be related to the evaluation time. In the present study, the evaluation was performed 37.2 ± 11.17 days after surgery. Post-transplant arterial hypertension seems to be related to an imbalance in water and sodium balance, vascular stiffness, endothelial dysfunction, abnormal cardiorenal neural reflexes resulting from immunosuppression, and cardiac denervation. Nephrotoxicity induced by calcineurin inhibitors and steroid use also contribute to post-transplant arterial hypertension^26^.

Arterial hypertension, as well as diabetes mellitus and dyslipidemia, should be screened for an early increase in arterial stiffness in heart transplantation patients, due to its association with cardiovascular events, increased mortality^14^, and vascular graft disease^28^. Vascular graft disease may be defined as a rapidly progressive proliferative disorder in the intimal layer of epicardial and intramural vessels, marked by smooth muscle proliferation, and accumulation of inflammatory cells and lipids, causing circumferential intimal thickening^29^. Vascular graft disease is the main cause of morbidity and mortality after 1 year of heart transplantation and accounts for approximately 30% of all-cause deaths in this population^30,31^. In this scenario, outpatient monitoring of cardiovascular parameters, obtained using a non-invasive method, could be tested as an adjuvant tool in the evolutionary analysis of vascular stiffness, considering the potential contribution of this phenomenon to the occurrence of vascular graft disease. Park et al.^28^ demonstrated a significant association between aortic cPP and the progression of vascular graft disease (*p* = 0.045). In the present study, cPP was significantly lower in the group of transplant candidates compared to the control group. Comparing the pre- and post-transplant cPP assessments, there was an increasing of 14.31% in this measure post-transplant, but without statistical significance.

Donor characteristics have also been associated with high blood pressure after heart transplantation, such as preexisting high blood pressure in the donor, older donor, male donor, and donor-recipient size mismatch. Although most heart recipients have lower blood pressure and cardiac output at the time of transplantation, many of them have already longstanding preexisting hypertension. This can result in chronic changes, including loss of vascular compliance and stiffness, further contributing to post-heart transplantation hypertension^26^.

As it is expected, cardiac output increased significantly in the postoperative evaluation, probably due to the concomitant increase in heart rate and stroke volume. A study in healthy young people demonstrated that the measures of systolic volume and cardiac output evaluated by the Mobil-O-Graph offer a good agreement of these parameters evaluated by echocardiography, which may bring perspective to the use of this method as an auxiliary tool in the monitoring of cardiac function in transplant patients^32^.

PWV, the gold standard for estimating arterial stiffness, increased by 11.56% postoperatively. Arterial stiffness modifies the properties of the large arteries, mainly causing a reduction in their ability to accommodate the blood ejected by the left ventricle during ejection. Structurally, changes in arterial stiffness occur in the long term, as in aging. On the other hand, short-term changes are related to mean arterial pressure, LVEF, or heart rate^33^ which in our study increased respectively by 21.18%, 143.85%, and 17.66%. Δ PWV strongly positively correlated with Δ pPAM (r = 0.9685, *p* < 0.0001), Δ cSBP (r = 0.9803, *p* < 0.0001) and Δ systolic volume (r = 0.7724, *p* < 0.0147). Δ LVEF or Δ heart rate were not associated with PWV.

In the present study, the AIx@75 increased by 18.19% after transplantation. However, this increase did not reach statistical significance, most likely due to the high variation coefficient of this variable. AIx@75 is an indirect indicator of arterial stiffness and an important predictor of cardiovascular events being considered a direct indicator of left ventricular afterload^34^. The relationship between ventricular compliance and contractility with changes in left ventricular afterload, called ventriculoarterial coupling, is poorly known. Latus et al. characterized systemic arterial elastance and its impact on left ventricular function and ventricular-arterial coupling in a cohort of children, adolescents, and young adults after heart transplantation. These authors observed that patients submitted to heart transplantation present impaired ventricular-arterial coupling at rest and in response to inotropic stimulation, despite having preserved left ventricular contractile reserve. An abnormal response in vascular function leading to an increase in afterload appears to be a significant factor that may play a role in the development of late graft failure^35^.

The pre- and postoperative functional capacity of patients who received heart transplantation was assessed using the DASI, and the score remained low in both moments. The DASI measures functional capacity and assesses aspects of quality of life. This questionnaire was developed from a study on peak oxygen consumption (peak VO_2_) during a maximal exercise test, therefore it addresses areas of functional capacity affected by heart disease^25^. DASI score < 34 (peak VO_2_ 17 to 18 mL/kg^−1^/min^−1^) can be used to identify patients at high risk of myocardial injury/infarction, moderate to severe postoperative complications, or new postoperative disability^6^. Buendia et al. compared the progression of exercise capacity and quality of life of patients able to complete a stress test 2, 6, 12, and 24 months after heart transplantation. Functional capacity, measured in METs and exercise time, progressively improved^5^. In the present study, the DASI score before and after heart transplantation was 14.32 ± 7.88 ml kg^−1^ min^−1^ and 13.18 ± 7.07 ml kg^−1^ min^−1^, respectively. The probable factor associated with the maintenance of low functional capacity in the studied population was the average length of hospital stay (≈37 days) after transplantation, mainly due to infectious complications (70%). Transplanted patients have a higher incidence of infectious conditions due to immunosuppression regimens^36^.

### Comparison of vascular, hemodynamic and arterial stiffness parameters in heart transplant candidates and control group

Peripheral and central pressure values were significantly lower in the pre-transplant group compared to the control group. Part of these changes can be attributed to the low LVEF presented by patients who are candidates for transplantation (27.54 ± 9.70%)^37–39^.

In the present study, we observed that the arterial stiffness indices of the patients in the transplant candidate group were similar to the control group in the preoperative period. The absence of changes in arterial stiffness indices may be related to the etiology of heart failure, most of which is non-ischemic, or to the effects of optimized clinical treatment for heart failure, with a greater frequency for the use of beta-blockers (91,7%). This class of drugs has vasodilator properties that can reduce systemic vascular resistance, and its chronic effects act by inhibiting neurohumoral responses that aggravate heart failure^40^. The reduction in systemic vascular resistance of this class of drugs counteracts the effects of calcineurin inhibitors on vascular smooth muscle^26^.

The present study also showed a significant reduction in the functional capacity of 24 patients who were candidates for heart transplantation, when compared to the control group matched by sex and age. The reduction in the functional capacity of patients with advanced heart failure is in line with the impact of the disease on the cardiopulmonary performance of these individuals^25,41^. Furthermore, this result corroborates the use of the DASI as a tool for assessing the functional capacity and risk stratification of candidate patients for heart transplantation^41,42^.

In the analysis of correlations, a positive association was found between AIx@75 and pulmonary artery systolic pressure (PASP) in candidates for heart transplantation. The evaluated patients had a mean PASP of 40.01 ± 12.28 mmHg. The functions of the left and right ventricles are closely linked. Elevated pulmonary artery pressure increases right ventricular afterload, and overall contractile performance becomes increasingly dependent on systolic ventricular interaction. In the presence of increased left ventricular end-diastolic pressure due to heart failure due to right ventricular systolic dysfunction, pulmonary artery pressure becomes elevated, imposing an increase in afterload on the right ventricle. In these patients, the global performance of the left ventricle is reduced, consequently, the systolic ventricular interaction is decreased, resulting in a reduction in the contractile performance of the right ventricle. In addition to the systolic interaction of the left ventricle with the right ventricle, when the right ventricle is enlarged and stretches the pericardium, pericardial and right ventricular diastolic pressures can increase markedly, resulting in restriction of filling of the left ventricle by the pericardium and the right ventricle via interventricular the septum^43^.

In the present study, pulse pressure amplification was significantly higher in the group of transplant candidates when compared to the control group. Pulse pressure amplification is a physiological phenomenon, characterized by the gradual increase in resistance from the aorta/carotid arteries to the brachial/radial arteries^44^. The pulse pressure amplification is calculated by the relation between the peripheral pulse pressure and the central pulse pressure and its reduction occurs in the presence of arterial stiffness. The increase in pulse pressure amplification in the present study can be attributed to the lower value of the reflection wave, assessed by the augmentation pressure (− 22.50%), and higher heart rate (11.98%) in the group of candidates for the heart transplantation compared to the control group. The reflection wave is modulated by the heart rate due to the relative time of the reflected wave in relation to the cardiac cycle, that is, the shorter the duration of the cardiac cycle, the smaller the reflection wave will be^45^.

Studies that point to arterial stiffness as a promising marker of cardiovascular diseases have gained particular importance after the development of the recent non-invasive and operator-independent techniques that allow the assessment of the main indices of arterial stiffness in a practical way. It is known that these indeces, especially AIx@75, can be influenced by medications, such as vasodilators and other antihypertensive drugs^46^. Therefore, more studies in larger populations are necessary to evaluate in detail how these external factors influence arterial stiffness indeces in order to improve the applicability of these indices in cardiovascular diagnosis. These further studies could contribute to the elaboration of possible correction factors that could be used to improve the assertiveness of these methods.

### Study limitations

The sample size is the main limitation of our study. These patients had different degrees of complexity, representing a heterogeneous population with regard to the level of severity, which may have impacted the evaluated parameters. The small sample made it impossible to assess the effects of different classes of drugs on cardiovascular parameters. Several drugs have been shown to influence arterial stiffness, including antihypertensive, statins, oral antidiabetics, glycation end-products (AGE) cross-link breakers, anti-inflammatory drugs, endothelin-A receptor antagonists, and vasopeptidase inhibitors. Only AGE cross-link breakers act directly on the structural component of arterial wall remodeling. Most of them act mainly on the dynamic component of arterial stiffness and, to a lesser extent, on the structural component^47^. In addition, we had to exclude patients using vasoactive drugs due to the great interference of these substances on hemodynamic and arterial stiffness results. The majority of critically ill patients to be transplanted have great hemodynamic instability and use these medications.

The subjective assessment of functional capacity can also be considered a limitation of the study. Cardiopulmonary exercise testing is considered the gold standard for assessing functional capacity. Around 80% of heart transplant patients at Santa Casa de Belo Horizonte have their procedure carried out urgently, being admitted in a serious condition, via hospital transfer, using dobutamine: for these patients it is neither possible nor necessary to perform the ergospirometry to indicate heart transplantation. Patients admitted on an outpatient basis are also frequently very seriously ill, with functional capacity grade IV and with predictors of poor prognosis with unequivocal indication for heart transplantation. In rare cases, in which clinical, laboratory evaluation and imaging tests are not sufficient to define the indication for transplantation, ergospirometry is performed at a Pulmonary Clinic affiliated with Santa Casa Hospital. Our hospital does not yet have this service, due to the high cost and also the need for qualified labor.

In our study, 78% of patients who were candidates for transplantation were men. Similar results were found by Chung et al. (2023). In view of this, we consider it a limitation of the study^48^.

## Conclusion

The low ejection fraction, systolic volume, and central and peripheral arterial pressures in the transplant candidate group may have negatively influenced the individual's functional capacity, as observed by the lower DASI score. Despite the significant increase in peripheral and central vascular pressures after surgery, the patients were normotensive. The 143.85% increase in LVEF in the postoperative period was not capable of positively affecting the functional capacity assessed by the DASI.

## Data Availability

The datasets used and/or analysed during the current study available from the corresponding author on reasonable request.
